# Evaluation of the availability of single‐position treatment with a rotating gantry and the validity of deformable image registration dose assessment for pancreatic cancer carbon‐ion radiotherapy

**DOI:** 10.1002/acm2.14330

**Published:** 2024-03-13

**Authors:** Yuya Miyasaka, Shohei Kawashiro, Sung Hyun Lee, Hikaru Souda, Mayumi Ichikawa, Hongbo Chai, Miyu Ishizawa, Takuya Ono, Hiraku Sato, Takeo Iwai

**Affiliations:** ^1^ Department of Heavy Particle Medical Science Yamagata University Graduate School of Medical Science Yamagata Japan; ^2^ Department of Radiation Oncology Kanagawa Cancer Center Yokohama Japan; ^3^ Department of Radiology Yamagata University Faculty of Medicine Yamagata Japan

**Keywords:** carbon‐ion therapy, deformable image registration, dose evaluation, pancreas cancer, treatment planning

## Abstract

**Background:**

This study aimed to evaluate the clinical acceptability of rotational gantry‐based single‐position carbon‐ion radiotherapy (CIRT) to reduce the gastrointestinal (GI) dose in pancreatic cancer. We also evaluated the usefulness of the deformable image registration (DIR)‐based dosimetry method for CIRT.

**Material and methods:**

Fifteen patients with pancreatic cancer were analyzed. The treatment plans were developed for four beam angles in the supine (SP plan) and prone (PR plan) positions. In the case of using multiple positions, the treatment plan was created with two angles for each of the supine and prone position (SP + PR plan). Dose evaluation for multiple positions was performed in two ways: by directly adding the values of the DVH parameters for each position treatment plan (DVH sum), and by calculating the DVH parameters from the accumulative dose distribution created using DIR (DIR sum). The D_2cc_ and D_6cc_ of the stomach and duodenum were recorded for each treatment plan and dosimetry method and compared.

**Results:**

There were no significant differences among any of the treatment planning and dosimetry methods (*p* > 0.05). The DVH parameters for the stomach and duodenum were higher in the PR plan and SP plan, respectively, and DVH sum tended to be between the SP and PR plans. DVH sum and DIR sum, DVH sum tended to be higher for D_2cc_ and DIR sum tended to be higher for D_6cc_.

**Conclusion:**

There were no significant differences in the GI dose, which suggests that treatment with a simple workflow performed in one position should be clinically acceptable. In CIRT, DIR‐based dosimetry should be carefully considered because of the potential for increased uncertainty due to the steep dose distributions.

## 1 BACKGROUND

Pancreatic cancer is one of the most intractable cancers.^1^ Among the treatment options, carbon‐ion radiotherapy (CIRT) reportedly has shown some efficacy in unresectable and recurrent cases.^2^, ^3^, ^4^, ^5^, ^6^ In CIRT for pancreatic cancer, the dose to the gastrointestinal (GI) tract surrounding the pancreas should be considered. In some cases, reducing the tumor dose is necessary to reduce the GI dose. With the introduction of a superconducting rotating gantry to the CIRT equipment,^7^ the optimum beam angle can be easily selected. Koom et al. reported the effectiveness of using a rotating gantry for the treatment of pancreatic cancer with CIRT.^8^ They demonstrated that a treatment plan with a rotating gantry could provide lower duodenal doses than those of a treatment plan with a fixed port and selectable beam angle while maintaining clinical target volume (CTV) coverage. In contrast, most CIRT equipment uses fixed ports,^9^, ^10^ which require multiple body positions to use multiple beam angles.^2^ A possible advantage of using multiple positions is that the GI shape and position may change at each position. Kim et al. evaluated pancreatic variations in the supine and prone patient positions and reported that the pancreas was particularly variable in the craniocaudal direction or that it differed depending on the body position.^11^ This finding suggests that multiple body positions may reduce the GI dose. If it is possible to reduce the GI dose, treatment in multiple body positions may also be considered when using a rotating gantry system. Miki et al. compared GI doses in treatment planning for different body positions during CIRT.^12^ However, there is currently insufficient evidence supporting the choice of either a single or multiple body positions when treating pancreatic cancer with CIRT.

Another problem with using multiple body positions is organ deformation, which makes high‐precision dosimetry difficult to achieve. One way to overcome this problem is to directly add up the dose–volume histogram (DVH) parameter values calculated for each body position. However, because this approach is a worst‐case assumption for organ at risk (OAR) doses, it may encourage evaluation of excessively high GI doses and GI dose reduction to the point beyond which the tumor dose decreases. As a countermeasure to this problem, a dose evaluation method using deformable image registration (DIR) was proposed. Hirai et al. evaluated dose summation using DIR for CIRT treatment planning for prostate cancer and examined the differences between water‐equivalent path length (WEPL)‐based, intensity‐based, and target‐based DIR algorithms as well as the target and OAR doses for prostate cancer.^13^ Li et al. described dose summation using DIR in CIRT for pancreatic cancer and demonstrated the relationship between the accuracy of the three DIR algorithms and the target dose.^14^ They found that a dice similarity coefficient (DSC) accuracy ≥0.89 is required to provide steep gradients in the CIRT‐dose distribution. DIR is sometimes used to calculate the true dose, accounting for organ variability, but there are few reports on the validity of DIR‐dose assessment in CIRT.

This study aimed to evaluate the GI dose when multiple body positions are used in the treatment of pancreatic cancer with CIRT to determine the most effective position when treating pancreatic cancer. This evaluation potentially could demonstrate the clinical acceptability of single‐position treatment with a rotating gantry in CIRT for pancreatic cancer. Additionally, the validity of dose assessment using DIR with multiple body positions in the treatment of CIRT was evaluated.

## 2 MATERIALS AND METHODS

### 2.1 Patient selection

Fifteen patients with pancreatic cancer treated by CIRT between 2022 and 2023 were randomly selected and analyzed. The exclusion criteria were patients for whom four‐dimensional computed tomography (4DCT) in either the supine or prone position could not be performed due to respiratory instability. The patient characteristics are shown in Table 1. The institutional review board of our institution approved this study.

**TABLE 1:** Patient characteristics.

Patient #	Gender	GTV site	GTV volume Supine CT (cc)	GTV volume Prone CT (cc)	CTV volume Supine CT (cc)	CTV volume Prone CT (cc)
1	F	Head	9.1	11.9	145.8	149.8
2	M	Body	2.5	2.7	158.5	163.3
3	M	Body	4.8	4.3	144.6	156.8
4	F	Head	17.4	23.9	204.3	220.9
5	M	Body	12.9	14.7	177.6	184.6
6	M	Head	9.6	14.1	114.7	116.6
7	F	Body	3.0	3.8	163.5	170.1
8	F	Hook	8.2	13.6	109.9	107.1
9	M	Body	10.9	9.4	81.0	80.1
10	M	Tail	8.4	8.7	146.8	150.8
11	F	Body	22.4	28.9	174.2	170.2
12	M	Body	8.0	8.3	174.4	211.6
13	F	Head	6.0	6.4	145.8	151.1
14	F	Body	0.9	1.2	158.3	159.7
15	F	Tail	9.7	9.5	85.5	82.6

Abbreviations: CT, computed tomography; CTV, clinical target volume; F, female; GTV, gross tumor volume; M, male.

### 2.2 CT image acquisition

Treatment planning CT images were acquired using Aquilion One (Canon Medical Systems, Otawara, Japan). For all patients, CT images in both the supine and prone positions were acquired simultaneously. The immobilization devices were BlueBAG (Elekta AB, Stochholm, Sweden) and Hipfix thermoplastic positioning (CIVCO Medical Solutions, Iowa, USA), and the patient was immobilized in both supine and prone positions with both arms raised. Then, 4DCT with a volume scan with the patient in the supine and prone positions was performed. The 4DCT was divided into 10 segments of 1 respiratory cycle, with 0% given at the most inspiratory part of the cycle and 50% at the most expiratory part of the cycle. Each CT image was obtained during 0%–90% of the respiratory cycle. We used AZ‐733VI (ANZAI Medical, Tokyo, Japan) to monitor respiratory waveforms from the abdominal wall. The CT reconstruction size was 2 mm, tube voltage was 120 kV, and mAs value was 105 mAs.

### 2.3 Treatment planning

Of the 10 phases of 4DCT, CT images acquired in the 50% respiratory phase were used, and the gross tumor volume (GTV), CTV, stomach, duodenum, and spinal cord were contoured by a radiation oncologist. The GTV was the primary target. The CTV was defined as the GTV plus the neurplexus area and regional selective lymph node area. Because the purpose of this study was to focus on the effects of different body positions, the treatment plan was designed without considering the effects of respiratory motion. RayStation 10A (RaySearch Laboratories, Stockholm, Sweden) was used as the treatment planning system. In this study, the PTV was not set, and the robust optimization function of the RayStation was used to create an error‐aware treatment plan. The robust optimization function of the RayStation uses a min‐max optimization algorithm.^15^ Robust optimization parameters were set for the CTV, with a position uncertainty of 2 mm in all directions. The irradiation technique involved spot scanning using a full‐energy scanning method.^16^ The dose‐calculation grid was 2 mm, and a pencil‐beam dose‐calculation algorithm was used. The CT images in the supine and prone positions were used to create a treatment plan that used a rotating gantry in each position to obtain four beam angles. Treatment planning in the supine position used 0°, 150°, 180°, and 270° beam angles (SP plan). Beam angles of 0°, 90°, 180°, and 330° were used for treatment planning in the prone position (PR plan). Next, a treatment plan using both the supine and prone positions was developed. This plan used 0° and 270° beams in the supine position and 0° and 330° beams in the prone position (SP + PR plan). The microdosimetric kinetic model was used to calculate the relative biological effectiveness (RBE) dose.^17^, ^18^ The prescribed dose was 55.2 Gy (RBE) in 12 fractions for all plans.^2^ Each of the four beams of each treatment plan was assigned 4.6 Gy (RBE) × 3 fractions out of 12 fractions. The optimization parameters set for the CTV and GI for each beam angle were the same in all cases, and the number of optimization iterations was 100 for each beam. GI gas was not overridden in this study. The reason for this was to eliminate the influence of the type of gas displacement technique on the results of the analysis.

### 2.4 Dose evaluation

In this study, the D_95%_ of CTV for SP plan, PR plan, SP+PR plan was evaluated as a percentage of the prescribed dose. In addition, the D_2cc_ and D_6cc_ in the stomach and duodenum were evaluated. These DVH parameters have been evaluated in previous reports on CIRT for pancreatic cancer.^19^, ^20^ For the SP and PR plans, the DVH parameters were calculated for the region of interest (ROI) of the organs on each treatment plan CT image. In the SP + PR plan, the DVH parameters were calculated in two ways. The first method arithmetically added the DVH parameter values calculated for each of the supine and prone CT images (DVH sum). The second method used the cumulative dose distribution with the DIR (DIR sum) to calculate the GI dose. In the DIR addition, as a first step, the DIR was performed with supine CT as the reference image and prone CT as the moving image. The DIR algorithm used was a hybrid intensity and structure‐based DIR algorithm provided by the RayStation.^21^ The stomach and duodenum were used as the Controlling ROI. The deformation grid size was set to 0.25 cm, and a correlation coefficient was used for the similarity metric. DIR accuracy was evaluated by the Hausdorff distance (HD) and DSC for each deformation contour of the stomach and duodenum.^22^, ^23^ The deformation vector field (DVF) calculated by the DIR between images was applied to the dose distribution of the treatment plan created on the prone CT of the moving image. The deformed dose distribution that was deformed by DVF on the prone CT was then mapped to the supine CT, and the accumulative dose distribution was calculated on supine CT. The DVH parameters for the stomach and duodenum were calculated from the accumulative dose distributions on the supine CT image.

### 2.5 Statistical analysis

SPSS version 28 (SPSS, Inc, Chicago, Illinois, USA) was used for statistical analysis. The doses calculated by the four methods were evaluated for significant differences by repeated measurements and Bonferroni correction, as normality was confirmed by the Shapiro‐Wilk test.

## 3 RESULTS

### 3.1 DIR accuracy

**FIGURE 1:**
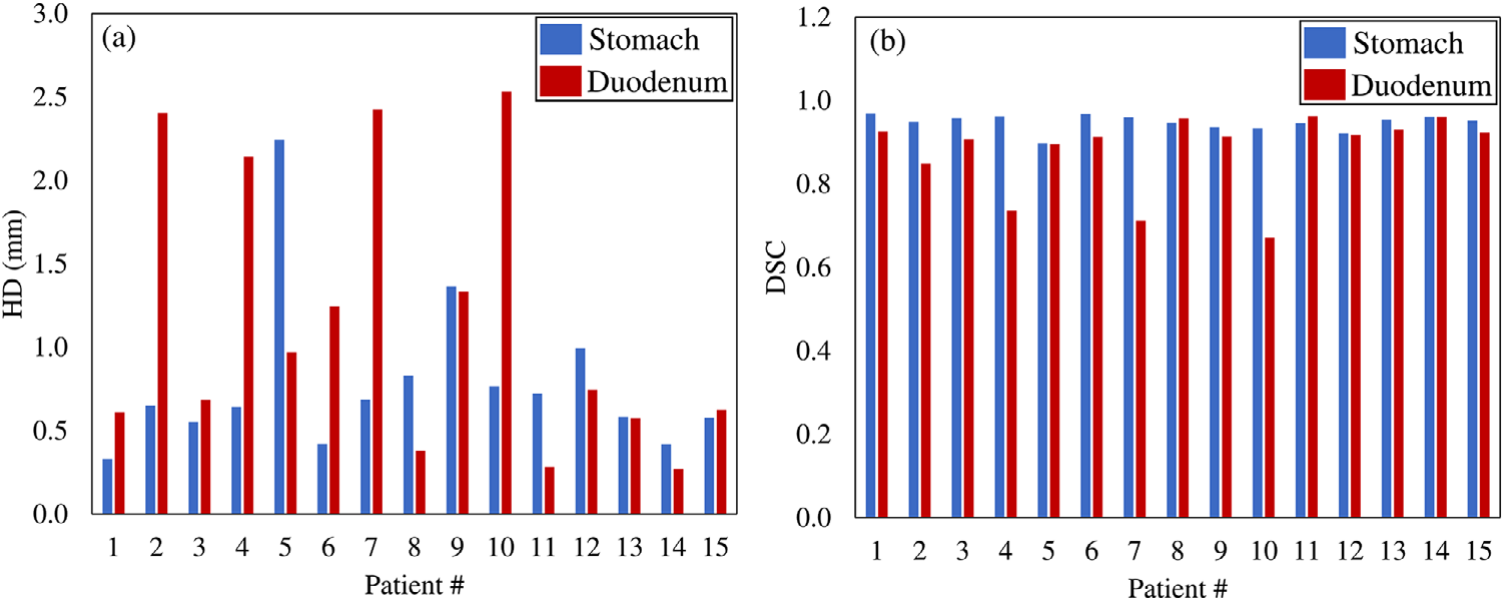
HD and DSC values for each case. DSC, dice similarity coefficient; HD, Hausdorff distance.

The mean ± SD of HD values in the stomach and duodenum were 0.79 ± 0.46 mm and 1.15 ± 0.80 mm, respectively, and the DSC values were 0.95 ± 0.02 and 0.88 ± 0.09. The HD and DSC for each case are shown in Figure 1, and the HD exceeded 2 mm in one case in the stomach and in four cases in the duodenum. The DSC was < 0.9 in no cases in the stomach and in four cases in the duodenum.

### 3.2 Dose assessment

The D_95%_ of CTV for the SP plan, PR plan, and SP+PR plan were 99.1 ± 0.2%, 99.1 ± 0.3%, and 99.0 ± 0.3%, with differences within 0.1%. The GI DVH parameter values for all cases are presented in Table 2. In the comparison of the SP and PR plans, the DVH parameters in the stomach were higher in the PR plan, and the DVH parameters in the duodenum were higher in the SP plan. However, there were no significant differences between the two treatment plans in either the stomach or duodenum. The DVH parameters for DVH sum ranged between the SP and PR plans for both the stomach and duodenum. In comparing the DVH and DIR sums, the DIR sum was higher at D_2cc_ and the DVH sum was higher at D_6cc_, although the differences were not significant.

**TABLE 2:** Mean DVH parameter values for all cases.

Organ	DVH parameter	SP plan [Gy (RBE)]	PR plan [Gy (RBE)]	SP + PR plan DVH sum [Gy (RBE)]	SP + PR plan DIR sum [Gy (RBE)]
Stomach	D_2cc_	29.5 ± 10.5 (23.5–35.4)	32.5 ± 6.3 (28.9–36.1)	31.4 ± 7.8 (27.0–35.9)	31.2 ± 9.4 (25.8–36.6)
	D_6cc_	20.1 ± 7.7 (15.7–24.5)	21.6 ± 6.9 (17.7–25.6)	21.4 ± 7.1 (17.3–25.5)	22.6 ± 7.9 (18.1–27.1)
Duodenum	D_2cc_	32.7 ± 7.2 (28.5–36.8)	29.6 ± 9.4 (24.2–35.0)	31.1 ± 8.3 (26.4–35.9)	30.5 ± 8.4 (25.6–35.3)
	D_6cc_	22.3 ± 7.9 (17.7–26.8)	20.4 ± 8.2 (15.7–25.2)	21.4 ± 8.0 (16.8–25.9)	22.2 ± 8.0 (17.6–26.7)

Organ	DVH parameter	*p*‐value SP plan vs. PR plan	*p*‐value SP plan vs. DVH sum	*p*‐value PR plan vs. DVH sum	*p*‐value DVH sum vs. DIR sum
Stomach	D_2cc_	0.777	0.388	1.000	1.000
	D_6cc_	1.000	0.392	1.000	1.000
Duodenum	D_2cc_	0.273	0.582	0.212	0.654
	D_6cc_	0.818	1.000	0.671	0.657

Values are mean+1SD (95% confidence interval).

Abbreviations: D_xcc_, minimum dose to the most irradiated x cc of tissue volume; DIR, deformable image registration; DVH, dose volume histogram; RBE, relative biological effectiveness.

**FIGURE 2:**
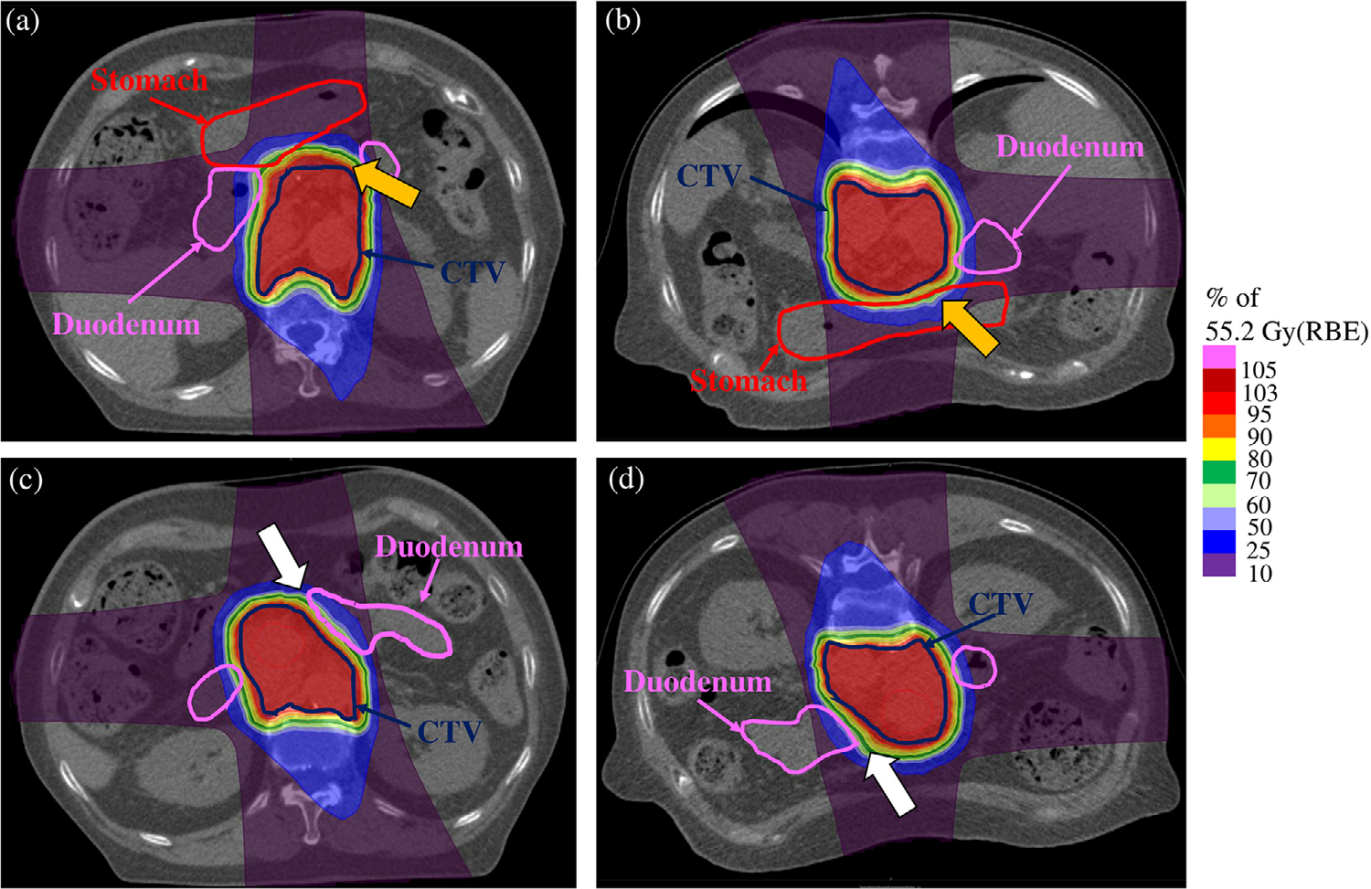
Dose distribution for patient 1. (a) and (c) represent SP plans, and (b) and (d) represent PR plans. For the stomach, in the PR plan shown in (b), compared to the RP plan shown in (a), the stomach is close to the CTV (yellow arrow) by pushing in the abdominal wall. For the duodenum, in the PR plan shown in (d) compared to the SP plan shown in (c), the abdominal wall is pushed in and the body spreads to the right and left, and the duodenum and CTV are separated (white arrows). CTV, clinical target volume; RBE, relative biological effectiveness.

The dose distributions of the SP and PR plans for patient 1 are shown in Figure 2. By comparing (a) supine CT and (b) prone CT, it was confirmed that the stomach was closer to the CTV in the PR plan. Comparison of the (c) supine CT and (d) prone CT in this case showed that the duodenum was close to the CTV on supine CT. The D_2cc_ values of the stomach and duodenum in the SP plan of this case were 21.5 Gy (RBE) and 37.9 Gy (RBE), respectively, whereas in the PR plan, the D_2cc_ values were 34.9 Gy (RBE) and 31.7 Gy (RBE).

**FIGURE 3:**
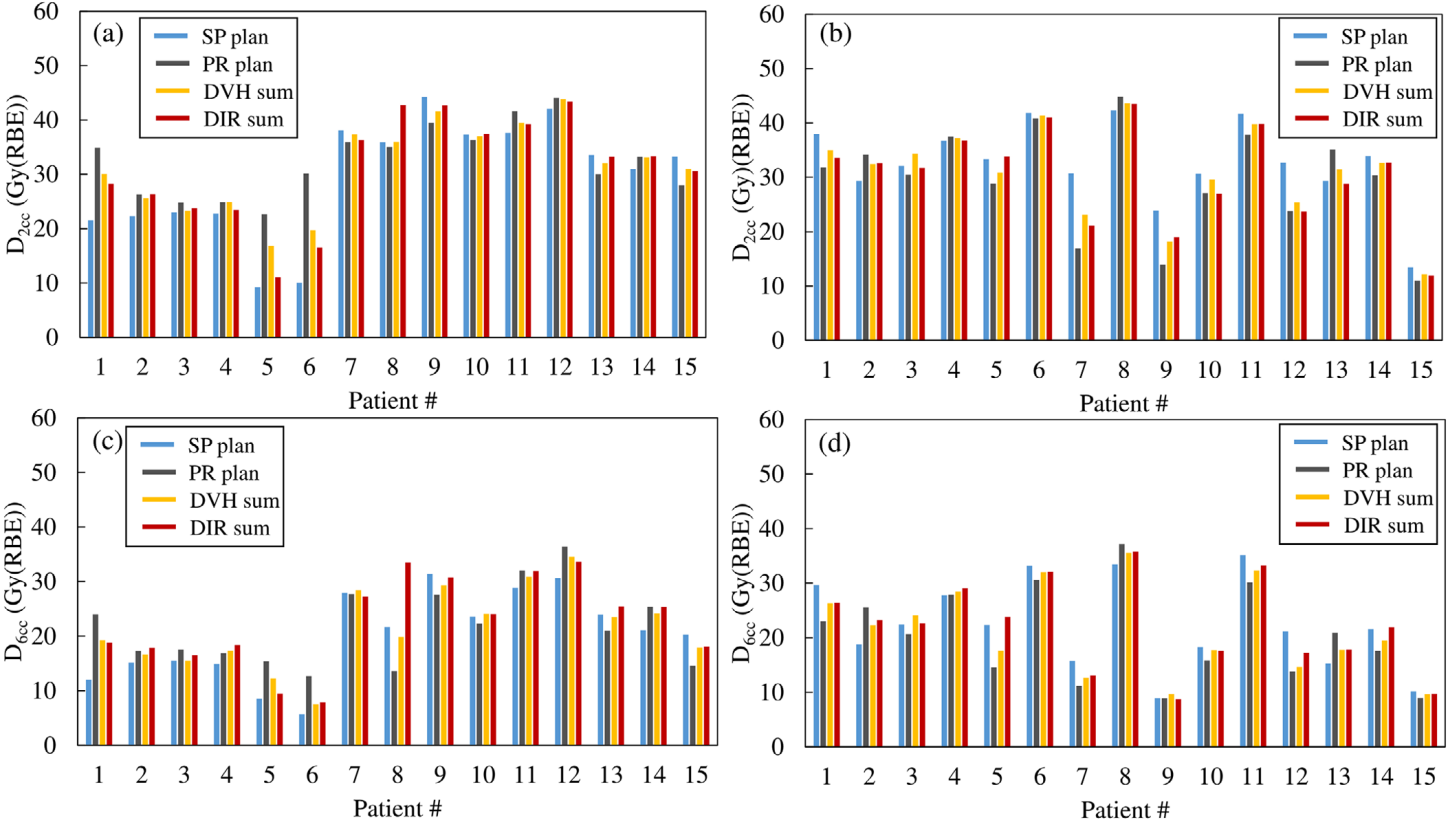
D_2cc_ and D_6cc_ of the stomach and duodenum in each case. (a) shows D_2cc_ of stomach, (b) shows D_2cc_ of duodenum, (c) shows D6cc of stomach, and (d) shows D_6cc_ of duodenum. DHV, dose volume histogram; DIR, deformable image registration; Dxcc, minimum dose to the most irradiated x cc of tissue volume; RBE, relative biological effectiveness.

Figure 3 shows the DVH parameter values for each treatment plan and accumulative dose‐calculation method for each case. The PR plan was higher in 9 of the 15 cases for both the D_2cc_ and D_6cc_ in the stomach. The duodenum was higher in the SP plan in 11 of the 15 cases. The DIR sum was higher than the DVH sum in 6 and 5 cases for D_2cc_ and in 9 and 11 cases for D_6cc_ in the stomach and duodenum, respectively.

**FIGURE 4:**
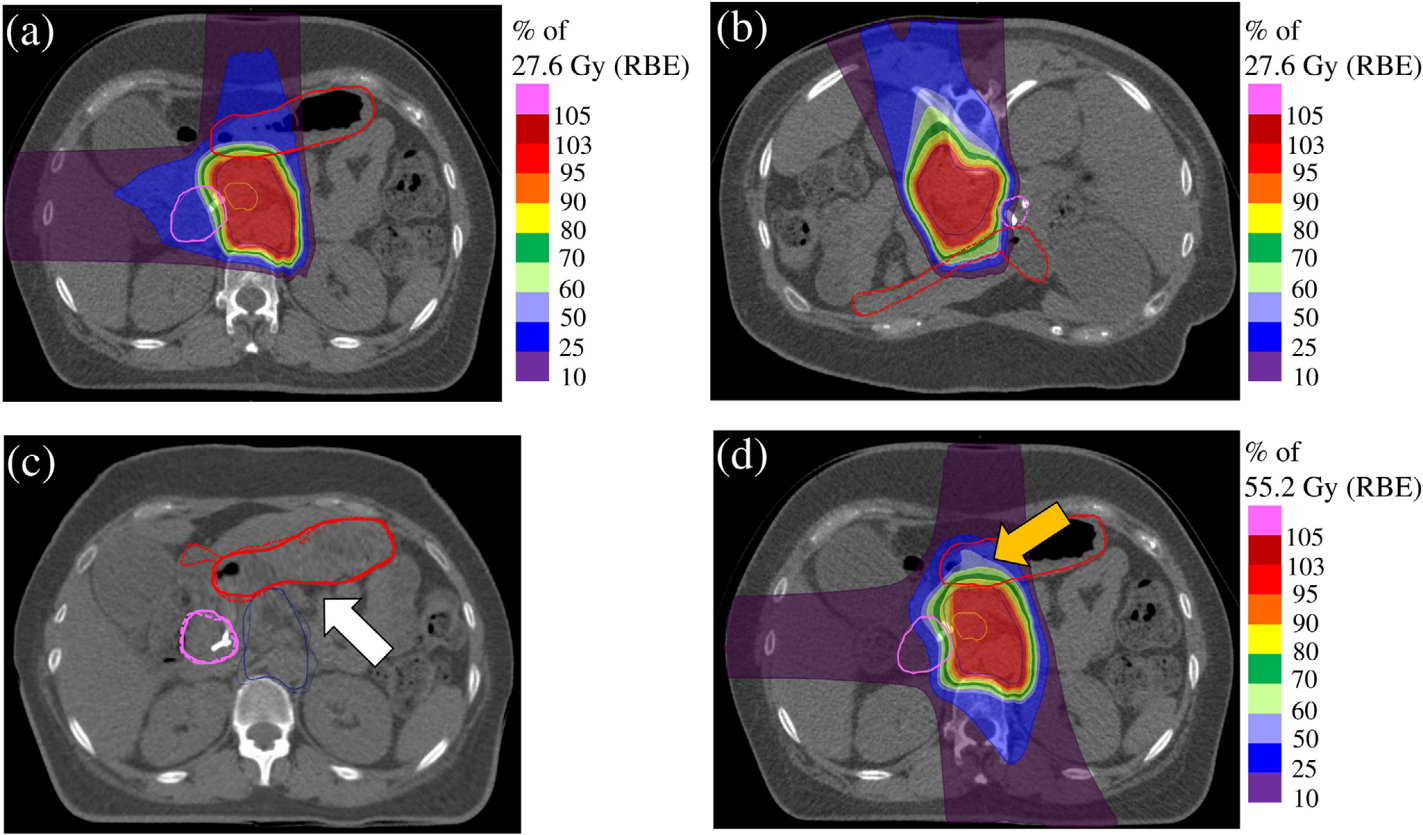
Reference CT image, moving CT image, and accumulative dose distribution formed by DIR. (a) is the supine CT, (b) is the prone CT, (c) is the CT image deformed by DIR, and (d) is an accumulative dose distribution. The CT image was deformed to match the ROI of the stomach, as shown by the yellow arrow. There was a broadening of the isodose line in the area indicated by the arrow in the accumulative dose distribution. CT , computed tomography; CTV, clinical target volume; DIR, deformable image registration; RBE, relative biological effectiveness; ROI, region of interest.

Figure 4 shows the dose distribution calculated using the DIR sum for patient 8. Compared with the stomach position in supine CT (a), the stomach position in prone CT (b) is pushed toward the pancreas. When DIR was performed, it confirmed that the contours of the stomach were well matched (c). In contrast, the accumulative dose distribution (d) shows that the 50%−60% isodose lines of the prescribed dose were widely distributed in the stomach. The D_6cc_ of the stomach in this case was 19.8 Gy (RBE) in the DVH sum and 33.5 Gy (RBE) in the DIR sum.

**FIGURE 5:**
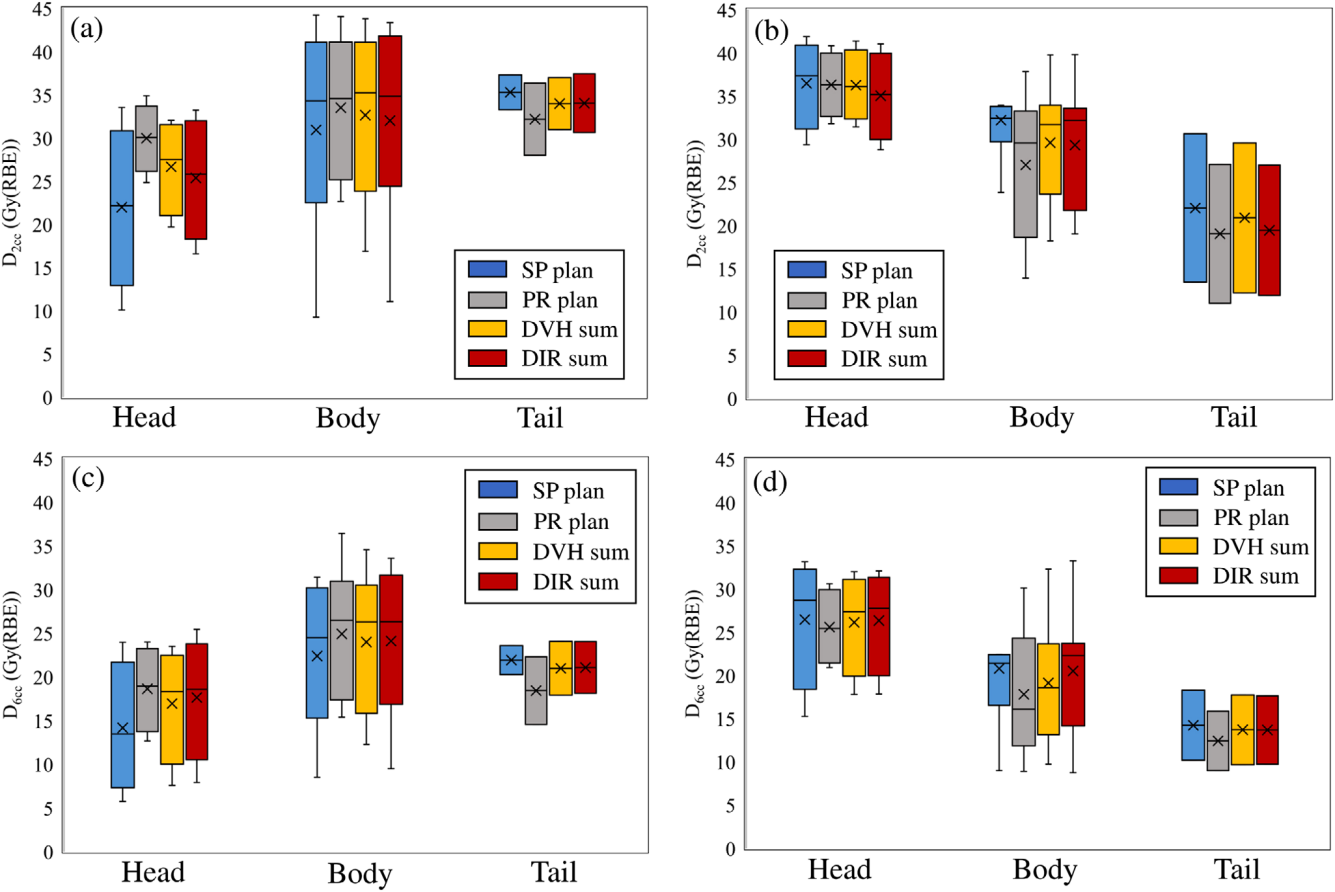
Box‐and‐whisker plot of DVH parameters for each GTV localization. (a) shows D_2cc_ of stomach, (b) shows D_2cc_ of duodenum, (c) shows D_6cc_ of stomach, and (d) shows D_6cc_ of duodenum. DIR, deformable image registration; DVH, dose volume histogram; GTV, gross tumor volume; D_xcc_, minimum dose to the most irradiated x cc of tissue volume; RBE, relative biological effectiveness.

The DVH parameters for each GTV localization are shown in Figure 5. Of the 15 cases, 4 were in the pancreatic head, 8 in the pancreatic body, and 2 in the pancreatic tail. Since there was only one case of a pancreatic hook, it was excluded and evaluated. Figure 5a and c shows that lower doses to the stomach in the pancreatic head cases were obtained with the SP plan than with the other treatment plans, but the difference between the treatment plans was smaller for the pancreatic body and pancreatic tail cases than for the pancreatic head cases. The duodenum doses shown in Figure 5b and d were lower for the PR plan in the pancreatic body cases than for the other treatment plans, but the difference between the treatment plans was small in the pancreatic head and tail cases.

## 4 DISCUSSION

In this study, we evaluated the GI dose in the treatment plan for each patient body position in pancreatic cancer CIRT. When both the supine and prone positions were used (SP+PR plan), the dose in the stomach was lower than that in the PR plan, and the dose in the duodenum was lower than that in the SP plan. However, there were no significant differences in either the stomach or duodenum doses for all treatment plans in all body positions. In addition, differences in CTV coverage parameters were within 0.1%. Therefore, the GI dose‐reduction effect using multiple body positions was limited, and it was considered acceptable to use only one position for CIRT. It is assumed that treatment in one position will reduce the uncertainty in the assessment of GI doses caused by organ deformations in multiple positions and allow for more accurate GI dose assessment.

Stomach doses were lowest when treatment was planned with the SP plan, and duodenum doses were lowest with the PR plan, although the mean comparisons were not significantly different. Miki et al. reported that the stomach dose was higher in the prone position than in the supine position, and the duodenum dose tended to be higher in the supine position than in the prone position.^12^ Figure 2 shows the dose distribution for this trend in GI doses. As shown in Figure 2b, the abdominal wall was pushed toward the pancreas in the prone position. Similarly, when the abdominal wall was pushed in the prone position, the duodenum, which surrounds the pancreas on the left, right, and bottom, was pushed to spread in each direction, as shown in Figure 2d, and the dose was considered to have decreased because of the distance created to the pancreas.

In the DVH sum for the SP+PR plan, the D_2cc_ and D_6cc_ of the stomach and duodenum showed values between those for the SP plan and PR plan. Although there was no significant difference, this trend suggests that both stomach and duodenum dose reductions can be achieved by using both the supine and prone body positions. This effect may also be because the dose for all beam angles for each body position in this study was 4.6 Gy (RBE) × 3 fractions. Depending on the location of the GI and tumor, some beam angles might reduce the GI dose more while guaranteeing a tumor dose at each beam angle. Koom et al. reported better angles, such as 90°, 45°, and 180° for the duodenum and 315° and 20° for the stomach, in terms of DVH parameters.^8^ Adjusting the prescribed dose for each beam angle may reduce the GI dose more effectively.

In this study, we compared the DVH sum and DIR sum as treatment planning dose assessment methods using two body positions. Regarding DIR accuracy, the results were similar to or better than the accuracy of the hybrid DIR in a similar study by Kubota et al.^24^ A comparison of the combined doses calculated by the DVH sum and DIR sum showed that the DVH sum was higher for D_2cc_ for both the stomach and duodenum. In theory, the DVH sum assumes a worst‐case scenario and should be higher than the DIR sum. From this point of view, the theoretical assumptions and results of this study are consistent with each other, and the evaluation of D_2cc_ using DIR generally provided a good estimation, but it was higher in D_6cc_ with the DIR sum, which might have been caused by errors introduced by the DIR. Figure 4 shows the accumulative dose distribution for patient 8. The stomach in the prone position shown in Figure 4b was deformed to match the stomach in the supine position shown in Figure 4a and to expand toward the abdominal wall shown in Figure 4c. This image deformation is thought to have deformed the dose distribution so that it spread toward the abdominal wall, resulting in a high dose distribution in the stomach, as shown in Figure 4d. The HD of the stomach in this case was 0.83 mm and the DSC was 0.95, showing generally good contour agreement by DIR. However, because the dose distribution may be excessively deformed, dose evaluation using DIR in CIRT requires both the value of the accuracy evaluation index and visual inspection of the dose distribution, with careful judgment. The HD exceeded 2 mm in the duodenum in four cases (patients 2, 4, 7, and 10) with respective DSCs of 0.85, 0.74, 0.71, and 0.67, which were lower than the overall mean value of 0.88. The DVH sum was greater than the DIR sum for all parameters except the D_2cc_ and D_6cc_ for patients 2 and 4 and the D_6cc_ for patient 7 in these four cases. The theoretical assumption that the DVH sum would be higher than the DIR sum was not warranted by the DIR accuracy evaluated by HD and DSC. Li et al. used DIR for pancreatic cancer CIRT and revealed the relationship between DIR accuracy and dose with respect to the accumulative tumor dose.^14^ Unlike in their study, no clear relationship between DIR accuracy and dose was necessarily stated in the peripancreatic GI doses evaluated in this study. In the dose assessment of CIRT using DIR, it is necessary to examine its validity through multifaceted evaluations, such as comparison of doses calculated according to the DVH sum and DIR sum, evaluation of DIR accuracy, and visual evaluation of the deformed or accumulative dose distribution.

In a previous study, Miki et al. evaluated GTV localization‐dependent doses of pancreatic cancer CIRT.^12^ They showed that for the stomach, the dose was higher in the pancreatic body and tail cases, and for the duodenum, the dose tended to be higher in the pancreatic head cases. This trend was similar to the trend in the present study. This trend is thought to depend on the extent of CTV target delineation due to the localization of GTV. In pancreatic head cases, the duodenal dose near the pancreatic head is higher, whereas in pancreatic body and tail cases, the CTV extends from the midline to the left side, resulting in a higher stomach dose. In this study, the proportion of dose reduction was larger in the supine position stomach for the pancreatic head tumors than for the body and tail, and the proportion of dose reduction was larger in the duodenum in the prone position in the pancreatic body tumors. Therefore, using the supine position for pancreatic head tumors may be advantageous for stomach dose reduction, and the use of the prone position for pancreatic body tumors may be advantageous for duodenum dose reduction.

Dose calculation accuracy is a crucial factor in CIRT dose assessment. The pencil beam algorithm used in this study has been used clinically,^25^, ^26^, ^27^ and based on previous treatment results, the dose error is considered acceptable.^3^, ^6^ In recent years, Monte Carlo algorithms have become available in commercial treatment planning system for particle therapy dose calculations.^28^, ^29^ It may be useful to revalidate this study with a more accurate dose calculation algorithm. In addition, DIR accuracy is an extremely important factor in dose assessment using DIR. The DIR algorithm used in this study is an algorithm that uses both image intensity and ROI information,^21^ and its accuracy has been verified in various studies.^30^, ^31^, ^32^, ^33^ In the current study, for example, the average HD and DSC of the stomach were 0.79 ± 0.46 mm and 0.95 ± 0.02, respectively, comparable to the accuracy of previous studies.^14^, ^24^ Because of the steeper dose distribution gradient of carbon beams, CIRT may require a higher DIR accuracy than x‐ray therapy, but its definite impact is not clear and should be carefully validated for clinical use. Further detailed studies on the relationship between CIRT dose distribution and DIR accuracy are expected.

There were several limitations in this study. First, the gantry angle was the same for all cases. A more optimal gantry angle may exist in some cases. Several previous reports have suggested optimal gantry angles.^8^, ^34^, ^35^ Depending on the combination of body position and gantry angle, there may be additional optimal combinations for the tumor dose that will be considered for further study. This study also did not include the effects of respiratory motion, and it is possible that these effects might be different between the supine and prone positions. The reason for not considering respiratory motion was to eliminate the effects of the various different respiratory motion treatment optimization methods from the results of the different treatment plans for each body position that should be the focus of this study. In addition, in this study, we did not override GI gas. The reason for this is also to focus on the results of differences in each body position, which is the purpose of this study. GI gas is a factor that varies greatly throughout the treatment period and can influence treatment.^36^, ^37^ Further study is required to determine the differences in the effects of GI gas in different body positions.

## 5 CONCLUSION

We compared GI doses in CIRT for pancreatic cancer between single position and multiple‐position treatment plans and investigated dosimetry methods for the multiple‐position treatment plans. Since there was no significant difference in the GI dose in any of the positions, treatment with a simple flow and a single position with a rotating gantry was considered acceptable in terms of the GI dose. However, since the use of multiple body positions tended to balance dose reduction across the entire GI, treatment using multiple body positions may be considered in some cases. Additionally, the DIR‐based dosimetry method may give an erroneous evaluation because of large errors depending on the DVH parameters to be evaluated, so more detailed studies are needed on the validity of DIR‐based dosimetry in CIRT.

## AUTHOR CONTRIBUTIONS

Yuya Miyasaka‐writing the manuscript, creating treatment plan, and data and statistical analysis; Shohei Kawashiro‐contouring ROIs, clinical integration, clinical review, and review manuscript; Sung Hyun Lee‐data analysis and review manuscript; Hikaru Souda‐data analysis and review manuscript; Mayumi Ichikawa‐contouring ROIs, clinical review, and review manuscript; Hongbo Chai‐review data and manuscript review; Miyu Ishizawa‐review data and review manuscript; Takuya Ono‐review data and manuscript review; Hiraku Sato‐clinical integration, clinical review, and review manuscript, Takeo Iwai‐management and coordination responsibility for the research activity planning and execution. Author responsible Q2 for statistical analysis is Yuya Miyasaka.

## CONFLICT OF INTEREST STATEMENT

The authors declare that they have no conflict of interest.

## Data Availability

Research data are stored in an institutional repository and will be shared upon request to the corresponding author.
